# Oxidized low density lipoprotein in the liver causes decreased permeability of liver lymphatic- but not liver sinusoidal-endothelial cells *via* VEGFR-3 regulation of VE-Cadherin

**DOI:** 10.3389/fphys.2022.1021038

**Published:** 2022-10-19

**Authors:** Alyssa R. Goldberg, Megan Ferguson, Sarit Pal, Rachel Cohen, David J. Orlicky, Rebecca L. McCullough, Joseph M. Rutkowski, Matthew A. Burchill, Beth A. Jirón Tamburini

**Affiliations:** ^1^ Department of Pediatrics, Section of Pediatric Gastroenterology, Hepatology & Nutrition. Children’s Hospital Colorado, Digestive Health Institute- Pediatric Liver Center, University of Colorado School of Medicine, Aurora, CO, United States; ^2^ Department of Medicine, Division of Gastroenterology and Hepatology, University of Colorado School of Medicine, Aurora, CO, United States; ^3^ Department of Pathology, University of Colorado Anschutz Medical Campus, Aurora, CO, United States; ^4^ Department of Pharmaceutical Sciences, Skaggs School of Pharmacy and Pharmaceutical Sciences, University of Colorado School of Medicine, Aurora, CO, United States; ^5^ Division of Lymphatic Biology, Department of Medical Physiology, Texas A&M University School of Medicine, Bryan, TX, United States; ^6^ Department of Immunology and Microbiology, University of Colorado School of Medicine, Aurora, CO, United States

**Keywords:** lymphatic endothelial cell, liver sinusoidal endothelial cell, alcohol-associated liver disease, cholestasis, non-alcoholic fatty liver disease, VE-cadherin, vascular endothelial growth factor, oxidized low density lipoprotein

## Abstract

The lymphatic vasculature of the liver is vital for liver function as it maintains fluid and protein homeostasis and is important for immune cell transport to the lymph node. Chronic liver disease is associated with increased expression of inflammatory mediators including oxidized low-density lipoprotein (oxLDL). Intrahepatic levels of oxLDL are elevated in nonalcoholic fatty liver disease (NAFLD), chronic hepatitis C infection (HCV), alcohol-associated liver disease (ALD), and cholestatic liver diseases. To determine if liver lymphatic function is impaired in chronic liver diseases, in which increased oxLDL has been documented, we measured liver lymphatic function in murine models of NAFLD, ALD and primary sclerosing cholangitis (PSC). We found that *Mdr2−/−* (PSC), Lieber-DeCarli ethanol fed (ALD) and high fat and high cholesterol diet fed (NAFLD) mice all had a significant impairment in the ability to traffic FITC labeled dextran from the liver parenchyma to the liver draining lymph nodes. Utilizing an *in vitro* permeability assay, we found that oxLDL decreased the permeability of lymphatic endothelial cells (LEC)s, but not liver sinusoidal endothelial cells (LSEC)s. Here we demonstrate that LECs and LSECs differentially regulate SRC-family kinases, MAPK kinase and VE-Cadherin in response to oxLDL. Furthermore, Vascular Endothelial Growth Factor (VEGF)C or D (VEGFR-3 ligands) appear to regulate VE-Cadherin expression as well as decrease cellular permeability of LECs *in vitro* and *in vivo* after oxLDL treatment. These findings suggest that oxLDL acts to impede protein transport through the lymphatics through tightening of the cell-cell junctions. Importantly, engagement of VEGFR-3 by its ligands prevents VE-Cadherin upregulation and improves lymphatic permeability. These studies provide a potential therapeutic target to restore liver lymphatic function and improve liver function.

## Introduction

Currently, the burden of chronic liver diseases is surpassing available therapeutics resulting in a rapid rise in the prevalence of advanced liver disease (GBD Cirrhosis Collaborators, 2020; Zhai et al., 2021). As a result, the number of patients that have progressed to cirrhosis, necessitating transplant, greatly surpasses the availability of donor organs (Kwong et al., 2022). Several chronic liver diseases that lead to cirrhosis are associated with high levels of oxLDL in the serum and liver including NAFLD (non-alcoholic fatty liver disease) (Burchill et al., 2021), HCV (hepatitis C virus), ALD (alcohol-associated liver disease) and experimental models of cholestatic liver disease (Alho et al., 2004; Comert et al., 2004; Schroder et al., 2006; Karadeniz et al., 2008; Ampuero et al., 2016; GBD Cirrhosis Collaborators, 2020).

The liver is the main producer and storage site for cholesterol, specifically low-density lipoprotein (LDL) (Suchy, 2020). As mentioned above, chronic liver diseases are associated with higher levels of the highly oxidized form of LDL. Endothelial cell induced conversion of LDL to oxidized LDL (oxLDL) leads to recognition of oxLDL by scavenger receptors (CD36, LOX1) expressed by many cells including hepatocytes, macrophages and endothelial cells (Itabe et al., 2011). OxLDL has been described to induce damage to liver parenchyma by way of macrophage activation (McGettigan et al., 2019), blood vascular endothelial injury (Sata and Walsh, 1998) and promotion of liver fibrosis (Karadeniz et al., 2008). In murine models of atherosclerosis, oxLDL has also been described to directly cause endothelial cell damage by inhibiting the expression of endothelial nitric oxide synthase (eNOS) (Di Pietro et al., 2016). OxLDL promotes the pathogenesis of atherosclerosis by inducing endoplasmic reticulum stress and cell death in vascular endothelial cells (Di Pietro et al., 2016). *In vitro*, oxLDL has a direct effect on human lymphatic endothelial cells (LEC) and results in more glycolytic LECs which downregulate the lymphatic transcription factor, *PROX1* (Burchill et al., 2021). These findings were consistent, based on gene expression data of liver LECs, in mice fed a high fat and high cholesterol diet (Burchill et al., 2021). Finally, oxLDL was able to directly cause a reduction in lymphatic permeability both in human LECs *in vitro* and a reduction of lymphatic drainage from the liver in mice injected with oxLDL (Burchill et al., 2021).

The consequences of increased oxLDL directly on the blood and lymphatic vasculature of the liver is still not well understood. Blood flows into liver acini predominantly *via* the liver sinusoidal endothelium. Liver sinusoidal endothelial cells (LSEC) have fenestrae which result in highly permeable sinusoids allowing for transcellular transport of fluid, proteins, and lipids (Braet and Wisse, 2002; Poisson et al., 2017). In the liver, the lymphatic capillaries are also highly permeable (Aukland et al., 1984; Chung and Iwakiri, 2013). The lymphatic capillaries are unique from blood vasculature in that their permeability comes from loose “button-like” junctions between endothelial cells comprised of VE-Cadherin, Claudin-5, Occludin and ZO1 (Baluk et al., 2007; Zhang et al., 2020). Both the lymphatic and the blood vascular endothelial cells in the liver can uptake and respond to oxLDL, but how these responses impact the progression of liver disease is yet unknown (Van Berkel et al., 1991; Singla et al., 2021). We and others have described differences in lymphatic function in response to oxLDL including decreased permeability and increased proliferation (Burchill et al., 2021; Singla et al., 2021). In contrast, oxLDL causes direct injury to the liver sinusoidal endothelium, leading to defenestration, by increasing reactive oxygen species, Nuclear Factor kappa B (NF-κB) activation, and caveolin expression (Zhang et al., 2014). Changes in LSECs were dependent on the LOX1 receptor (Zhang et al., 2014). OxLDL scavenger receptors include LOX1, CD36, SR-A1 and SR-B1, where CD36 and SR-B1, rather than LOX1, are predominantly expressed by LECs (Singla et al., 2021). Interestingly, CD36 expression is higher in LECs compared to human umbilical vein endothelial cells, while SR-B1 expression was equivalent accross endothelial cell types (Singla et al., 2021). These findings predict that there is differential signaling of oxLDL in blood versus lymphatic vascular endothelial cells.

Endothelial cells of blood and lymphatic lineage perform unique functions as a result of differential expression and signaling of vascular endothelial growth factor (VEGF) receptors. The lymphatic endothelium predominantly express and signal through VEGFR-3 homodimers *via* engagement of VEGF-C and D (Rauniyar et al., 2018). Signaling through VEGFR-3 promotes the expression of transcripts that are essential for lymphatic cell identity, homeostasis and proliferation, such as *Lyve1 and Prox1* (Deng et al., 2015). In contrast, the blood vasculature predominately utilizes signaling through VEGFR-2 which is maintained by VEGF-A and low levels of Neuropilin-1 (a scavenger receptor for VEGF-A) (Shin et al., 2008; Zhang et al., 2020). Further, the VEGFR-2 signaling pathway has been described to control endothelial cell permeability by regulating the expression of VE-Cadherin (Wallez et al., 2006). In blood endothelial cells (BEC) VEGFR-2 engagement results in phosphorylation of SRC, AKT upregulation and VE-Cadherin stabilization at the cell surface (Braet et al., 2004; Ding et al., 2010). While VEGFR-2 and VEGFR-3 have several common signaling modalities in endothelial cells, differences in VEGF receptor signaling in the regulation of VE-Cadherin in BEC versus LECs is still not well understood. Evidence that VEGFR-3 signaling in LECs is unique has been established using a mutant form of VEGF-C (cys156ser) which can only bind to VEGFR-3 homodimers (Breslin et al., 2007) predominantly expressed by LECs. Indeed, use of rVEGF-C cys156ser improves lymphatic transport from the liver in an experimental model of NASH (Burchill et al., 2021). Furthermore, VEGFR-2 signaling, as a result of VEGF-A administration, in the lacteals resulted in increased VE-Cadherin staining that more closely resembled tight “zipper” rather than loose “button” junctions (Zhang et al., 2018). This was in contrast to VEGFR-3 homodimer signaling *via* administration of rVEGF-C cys156ser which did not cause junctional zippering (Zhang et al., 2018). Thus, signaling through VEGFR-2 and VEGFR-3 differ in their ability to regulate VE-Cadherin surface expression in both blood vascular and LECs.

In this study we add to the understanding of lymphatic dysfunction in disease. We dissect the phenotypic and functional consequences of oxLDL exposure and VEGFR-3 signaling on both LEC and LSECs. We demonstrate that lymphatic drainage is impaired in multiple models of chronic liver disease, all of which have been reported to have increased intrahepatic oxLDL in humans. To determine the consequence of increased oxLDL on the LSECs and LECs in isolation we utilized an *in vitro* system where we found the permeability of lymphatic, but not sinusoidal endothelial cells was decreased in the presence of oxLDL. This decreased permeability was a direct result of increased VE-Cadherin levels, a likely result of increased SRC and MAPK activation in LECs but not LSECs. We further demonstrate that signaling through VEGFR-3 improved oxLDL induced changes in permeability through the manipulation of VE-Cadherin. Using an *in vivo* model of lymphatic transport, we demonstrate oxLDL causes reduced lymphatic transport of FITC-Dextran from the liver which can be rescued following overexpression of VEGF-D, a VEGFR-3 ligand, in the hepatic parenchyma. This recovery of lymphatic transport in the presence of VEGF-D was associated with decreased expression of VE-Cadherin on LECs from the liver*.* This study is significant because it suggests that the increased oxLDL in the liver of patients with chronic liver disease directly impedes lymphatic function, which is necessary for organ health.

## Results

### Reduced trafficking of high molecular weight dextrans to liver draining lymph nodes of mice with increased oxLDL receptor expression

**FIGURE 1:**
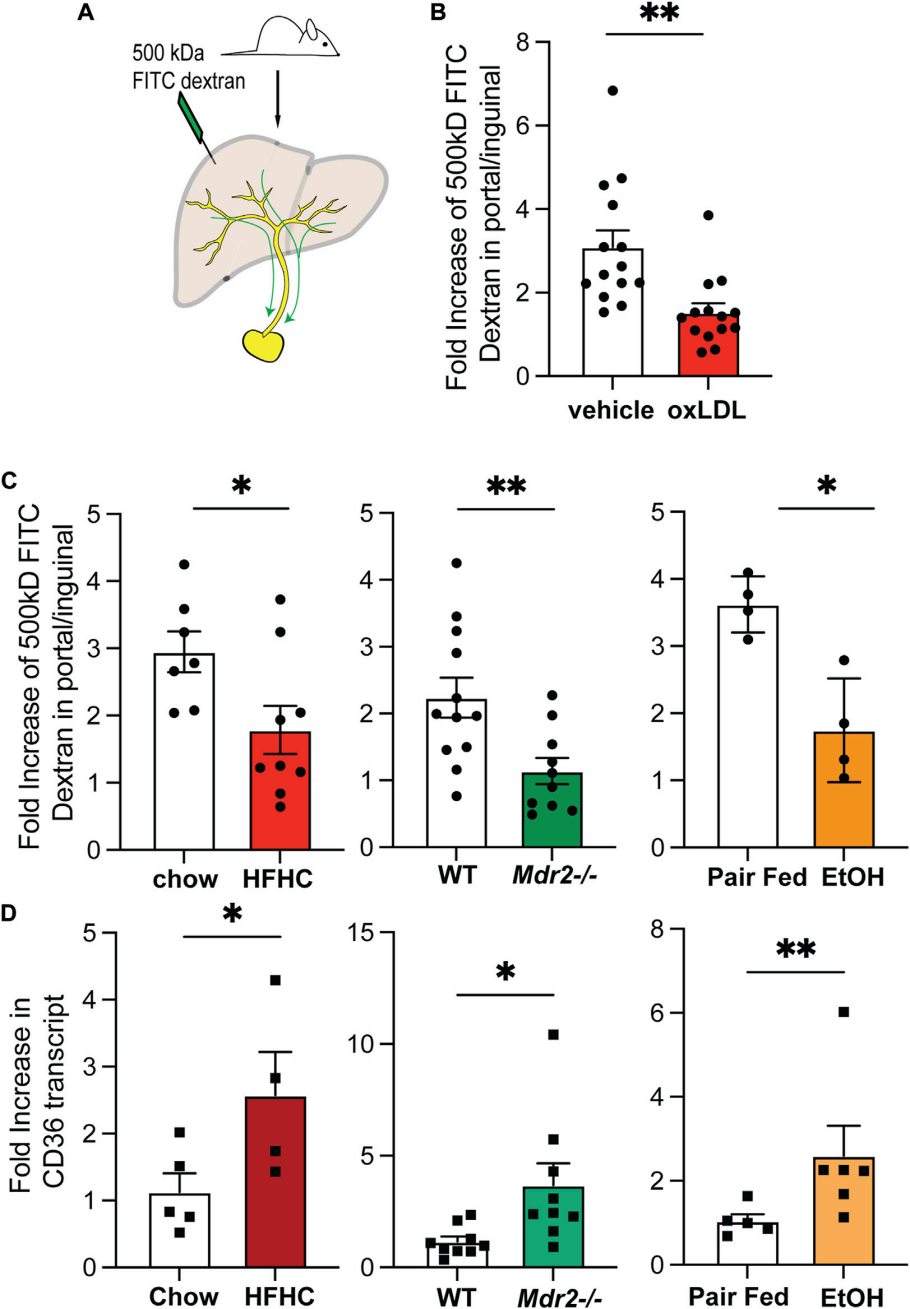
Reduced trafficking of high molecular weight Dextrans to liver draining lymph nodes of mice with increased oxLDL receptor expression. **(A)** Mice were anesthetized and abdominal cavity exposed. 10uL of 500 kDa FITC-dextran was injected each into the left, median and right lobes of the liver. Ten minutes after the last injection, the portal (liver-draining) and then inguinal (skin-draining) lymph nodes were extracted. **(B)** Quantification of FITC in portal compared to inguinal lymph nodes in mice treated with oxLDL (2-week course of 150 ug every other day) as compared to matched controls. Shown are 3 combined experiments with 3-5 mice per experiment. **(C)** Amount of FITC measured by fluorescence in the portal as compared to the inguinal lymph node per mouse in mice fed a 24-week HFHC (NAFLD model), mdr2 −/− mice (cholestasis model), or Lieber deCarli fed mice (ALD model) with respective matched controls. HFHC is 2 combined experiments with 4-5 mice per group. Mdr2−/− is 3 combined experiments with 3-5 mice per group. EtoH is representative data from one experiment performed twice. **(D)** CD36 expression by RTPCR of whole liver mRNA as compared to intra-experimental matched controls per experimental group. Shown are representative graphs of experiments repeated on 2-3 occasions with 3-5 replicates per time point with similar results. *p < 0.05, **p < 0.01, ***p < 0.001.

In our previous report we outlined that oxLDL directly affects the amount of high molecular weight FITC Dextrans in the liver draining lymph nodes. These studies were performed both in mice fed a diet consisting of 40% fat and 2% cholesterol over a 20-week period and following direct intravenous injection of oxLDL over a 2-week period (Burchill et al., 2021). We injected 500 kDa FITC labeled Dextran into the hepatic parenchyma in mice and assessed accumulation of the injected FITC in the liver-draining lymph node (LN) at 10 min post intrahepatic injection as previously described (Burchill et al., 2021) (Figure 1A). We controlled for systemic distribution of FITC by assessing FITC levels in the non-liver-draining inguinal lymph node (Burchill et al., 2021). As demonstrated previously, we observed a significant reduction in the amount of FITC in the liver draining lymph node following treatment with oxLDL at 10 min (Figure 1B). Whether lymphatic transport of high molecular weight Dextrans is impaired in other liver disease etiologies, also reported to have increase oxLDL within the serum, was not yet known. To address this question, we evaluated additional murine models including an ethanol containing Lieber-DeCarli diet model to mimic murine ALD (Guo et al., 2018) and an *Mdr2* ^*−/−*^ model of fibrosing cholangiopathy (Mariotti et al., 2018) (Supplementary Figure S1). To determine if the loss of high molecular weight FITC Dextran in the liver draining lymph nodes was independent of disease etiology we measured lymphatic drainage in the ALD and *Mdr2−/−* models and found that, relative to the control, less FITC-dextran reached the draining lymph node within 10 min (Figure 1C). This decreased lymphatic drainage was not due to reduced lymphatic vasculature in the liver as we have previously reported expansion with increased lymphatic vessel density (LVD) in these mice models (Burchill et al., 2021) and humans (Tamburini et al., 2019) with the corresponding disease. The differences in FITC-Dextran accumulation in the liver draining lymph nodes was similar to the difference in the amount of FITC-Dextran in the lymph nodes after intrahepatic injection in a high fat high cholesterol (HFHC) diet model (Figure 1C) and mice injected with oxLDL (Figure 1B). CD36 is well described as a scavenger receptor for oxLDL (Chen et al., 2008; Jay et al., 2015) and increased CD36 expression in the liver has been described as being associated with an increase in oxLDL (Feng et al., 2000). Consistent with increased oxidation of cholesterol we find increased CD36 expression in the livers of mice fed the HFHC diet for 24 weeks, given the Lieber-DeCarli diet and the *Mdr2* ^*−/−*^ model of cholestatic liver disease (Figure 1D). Together, these findings are consistent with increased oxLDL sensing associated with chronic liver disease across disease etiologies. This impairment in FITC trafficking to the liver draining lymph nodes suggests that decreased transport of high molecular weight molecules from the liver *via* the lymphatics is a common event in many chronic liver diseases associated with increased oxLDL.

### oxLDL increases VE-Cadherin surface expression and decreases permeability of LECs in contrast to LSECs

**FIGURE 2:**
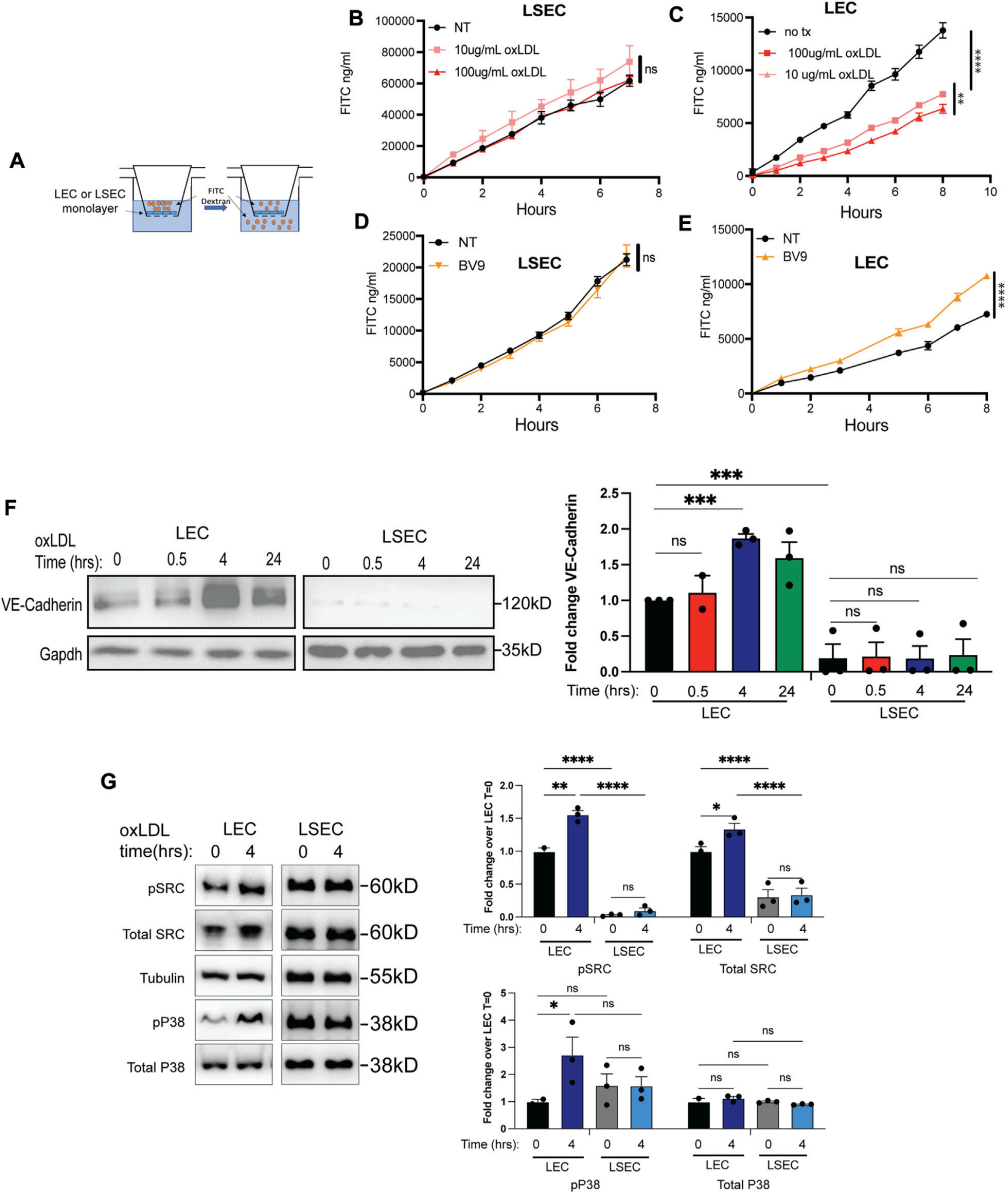
oxLDL increases VE-Cadherin surface expression and decreases permeability of lymphatic endothelial cells without affecting liver sinusoidal endothelial cell permeability. **(A)** Experimental model of 0.4 uM transwell with confluent layer of human LECs or LSECs after allowing them to grow to confluency. After 3 days, 1.25 mg/ml 500 kDa FITC-dextran added to the top chamber at start of time course and then amount of FITC migrated to bottom chamber measured hourly by fluorescence. **(B)** LSECs treated with 4 h of 10 or 100 ug/mL oxLDL in top chamber for 4 h prior to addition of FITC-dextran, shown is quantified amount of FITC in lower chamber per time-point. Linear regression performed and difference between slope of lines was not significant. Experiment was repeated 3 times with 3-4 replicates per group. **(C)** Transwell experiment with LECs treated with 4 h of 10 or 100 ug/mL oxLDL prior to addition of FITC-dextran values reported as quantified amount of FITC in lower chamber per timepoint. Linear regression performed and difference between slope of NT vs. 10 ug/mL and 10 ug/mL and 100 ug/mL was <0.0001 and 0.005 respectively. Experiment repeated 4 times with 3-4 replicates per group with similar results. **(D)** LSECs treated with same BV9 clone of VE-Cadherin for 4 h under same conditions as LECs with linear regression analysis revealing no significant difference between lines with 3 replicates per group two independent times with similar results. **(E)** LECs treated with BV9 clone of VE-Cadherin antibody for 4 h prior to addition of FITC-dextran as above, linear regression analysis revealed a significant difference between lines with a *p*-value of <0.0001 as demonstrated by 4 stars. This experiment was repeated twice with 3-6 replicates per group with similar results. **(F)** Protein expression of VE-Cadherin in LECs and LSECs treated with 100 ug/mL oxLDL for 0 min, 30 min, 4 h or 24 h in serum-free media. Western blot analysis using antibodies against VE-Cadherin and GAPDH (loading control). Experiment was performed 3 times (except 0.5 h time point for LECs which was repeated twice) and Western blot shows representative image with quantification from 3 replicates each divided by the GAPDH control and normalized to time 0. **(G)** Protein expression of total and phosphorylated SRC and P38 in cells treated with 100ug/mL oxLDL for 4 h in serum-free media. Western blot analysis using antibodies against pSRC, SRC, pP38, P38 and tubulin (loading control). Experiment was performed 3 times and representative image is shown with quantification from 3-4 independent experiments divided by tubulin and normalized to time 0. One-way anova with multiple comparisons was performed on Western blot quantification where *p < 0.05, **p < 0.01, ***p < 0.001.

As the protein content of the lymph draining the liver is influenced by LSEC and LEC permeability, we asked which endothelial cells were most affected by oxLDL in regard to permeability. We compared the transport of FITC-Dextran across a monolayer of either LECs or LSECs (Figure 2A). LECs have highly permeable cell-cell junctions to allow for paracellular transport of solutes and lymph, but also participate in transcellular transport over time and under the conditions of fluid flow (Triacca et al., 2017). In contrast LSECs have tighter less permeable junctions but are highly fenestrated (Braet and Wisse, 2002; Poisson et al., 2017). We found that over time, the FITC Dextran accumulated in the bottom well more quickly through the LSEC monolayer than the LEC monolayer (Figures 2B,C). Furthermore, treatment with oxLDL did not impact the ability of FITC dextran to migrate across the LSEC monolayer. In contrast, and similar to our previous report, we found that after a 4-hour treatment with oxLDL there was decreased permeability of the LEC monolayer which could be titrated based on the dose of oxLDL (Figure 2C). OxLDL results in the increased expression of VE-cadherin on LECs in a dose-dependent manner (Supplementary Figure S2A), therefore we examined the contribution of VE-Cadherin to cellular permeability of LSECs and LECs. Using an antibody which disrupts VE-cadherin dependent cell-cell junctions (BV9), we tested endothelial cell permeability. An LSEC monolayer was treated with the VE-Cadherin antibody and there was no significant difference in permeability compared to control (Figure 2D). In contrast, we found that blocking VE-Cadherin cell-cell junctions resulted in increased permeability of the LEC monolayer (Figure 2E). As previously demonstrated (Baluk et al., 2007) these findings confirm that LEC permeability relies on VE-Cadherin junctions and paracellular transport while LSEC permeability does not rely on VE-Cadherin junctions and may be more dependent on transcellular transport.

The differential role of VE-Cadherin on LECs compared to LSECs is further supported by total VE-Cadherin protein expression being markedly greater in LECs as compared to LSECs following treatment with oxLDL. When comparing equivalent amounts of total protein, the LECs showed significantly increased VE-Cadherin protein levels at 4 h after oxLDL (Figure 2F). In contrast, primary cultured LSECs have minimal VE-Cadherin protein expression in the presence or absence of oxLDL (Figure 2F). To evaluate the mechanism by which these 2 cell types have differential responses to oxLDL treatment, we asked whether intracellular signaling upstream from VE-Cadherin was different between cell types. We found that both SRC and P38 phosphorylation was increased after 4 h s of oxLDL treatment in LECs but not LSECs (Figure 2G and Supplementary Figure S2B). These findings suggest that the downstream signaling pathway from oxLDL in LECs is distinct from the signaling in LSECs and that these different signaling pathways correlate with distinct response in LECs compared to LSECs treated with oxLDL (Singla et al., 2021).

### Vascular endothelial growth factor C and D improve lymphatic specific changes caused by oxLDL

**FIGURE 3:**
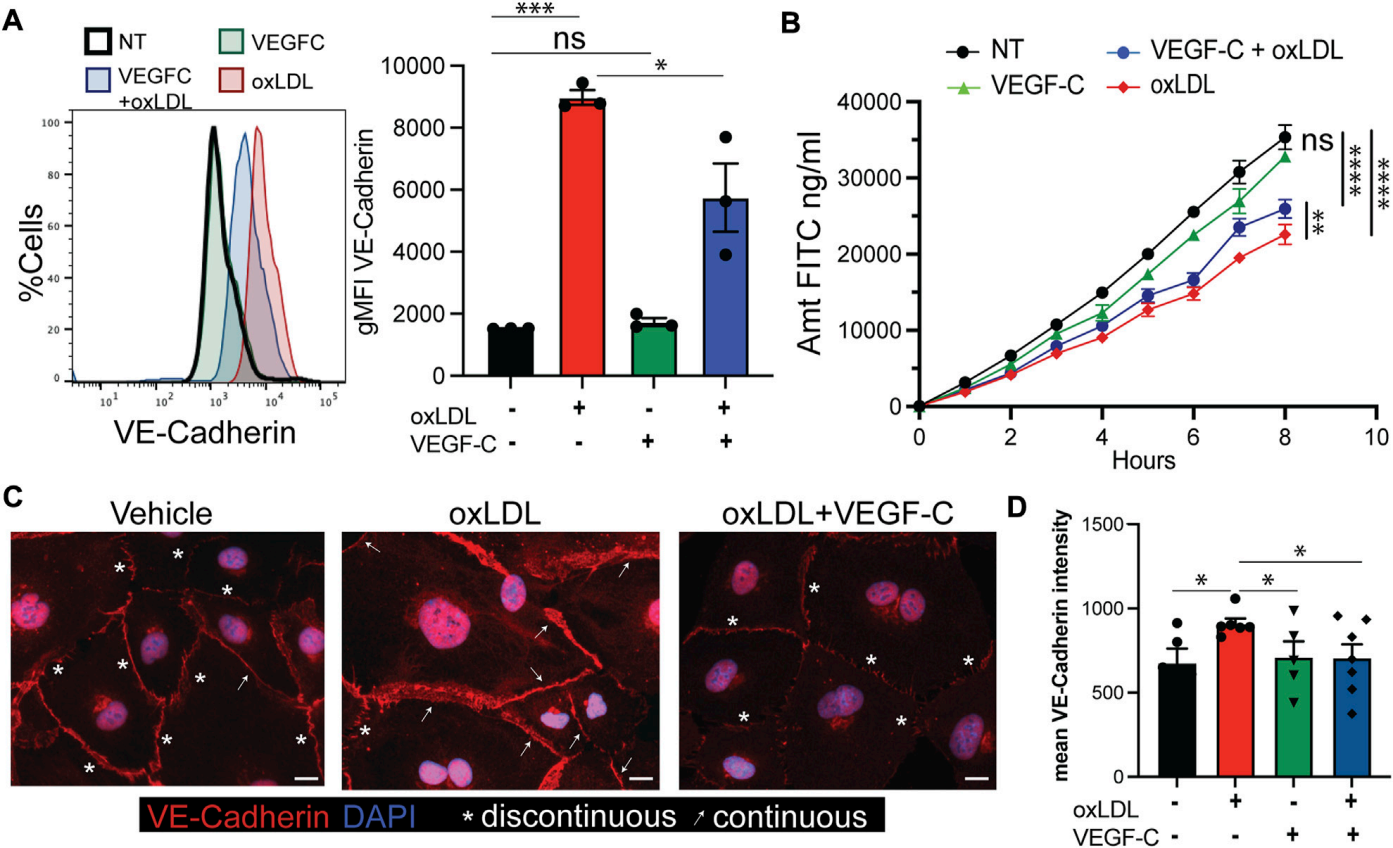
Vascular endothelial growth factor C and D reverse lymphatic specific changes caused by oxLDL. **(A)** Histogram of VE-Cadherin surface expression on LECs treated with PBS or VEGF-C (Cys^156^→Ser mutant) for 16 h prior to addition of oxLDL or PBS for 4 h before harvest and staining with BV9 clone of VE-Cadherin. Bar graph representative of gMFI of VE-Cadherin in LECs with above treatments and ANOVA analysis demonstrated in a significant difference between oxLDL and oxLDL + VEGF-C. This experiment was repeated three times with 2-3 replicates per group with similar results. **(B)** Transwell experiment with LECs plated to confluency as described, treated for 16 h with PBS or VEGF-C (Cys^156^→Ser mutant) then oxLDL or PBS for 4 h prior to addition of 1.25 mg/ml of 500 kDa FITC-dextran to the top chamber with measurement of FITC migrated to the lower chamber. Shown is quantified amount of FITC in bottom well. Linear regression performed and difference between slopes of oxLDL vs. oxLDL + VEGF-C groups was significant. Experiment was repeated two times with 3-4 replicates per group with similar results. **(C)** hLECs were plated, grown to confluence and treated with oxLDL ± VEGF-C as in **(A**,**B)**. Cells were stained with VE-Cadherin (Red), Dapi, blue and labeled based on junctional structures to have continuous (arrow) or discontinuous (asterisk) VE-Cadherin staining. **(D)** Quantification of mean fluorescence intensity of VE-Cadherin. Dots represent blindly selected individual regions of each slide in order to minimize differences in confluence, staining within slides and across slides. Experiment was repeated 2–5 times (depending on treatment) with 2–3 separate chambers per group with similar results. *p < 0.05, ***p < 0.001.

Signals derived through the VEGF receptors have been shown to regulate VE-Cadherin junctions (Zhang et al., 2018; Burchill et al., 2021). We previously demonstrated in a mouse model of NASH that liver LECs have increased expression of VEGFR-2 transcript and treatment of NASH mice with rVEGF-C (cys156ser), to induce signaling through VEGFR-3, improved lymphatic drainage from the liver (Burchill et al., 2021). To test whether we could manipulate VE-Cadherin transcript levels *via* engagement of VEGFR-3 we added exogenous recombinant (r)VEGF-D to oxLDL treated LECs. Interestingly transcript levels of VE-Cadherin (*CDH5*) in oxLDL treated LECs were reduced after addition of rVEGF-D (Supplementary Figure S3A). We also previously demonstrated that oxLDL treatment decreased the expression of *PROX1* in hLECs*,* the transcription factor required for lymphatic differentiation (Burchill et al., 2021). In contrast to VE-Cadherin, we did not detect differences in *PROX1* expression with the addition of rVEGF-D to oxLDL treated LECs (Supplementary Figure S3B). However, consistent with our previous report (Burchill et al., 2021), the VEGFR-2/3 [*KDR/FLT4*] ratio was increased after oxLDL treatment (Supplementary Figure S3C), skewing the oxLDL treated LECs toward VEGFR-2 signaling. After exposure to rVEGF-D in the setting of oxLDL, we found that the VEGFR-2:3 ratio was normalized, suggesting that signaling is shifted back to lymphatic-specific VEGFR-3 compared to VEGFR-2 (Supplementary Figure S3C). To directly connect VEGFR-3 signaling and VE-Cadherin expression, we next asked if oxLDL dependent upregulation of LEC membrane VE-Cadherin expression would be reduced by engaging VEGFR-3 homodimers with recombinant VEGF-C (cys156ser). We found cell surface expression of VE-Cadherin was reduced after treatment with rVEGF-C and oxLDL compared to oxLDL alone (Figure 3A). Based on the decreased expression of VE-Cadherin after treatment with rVEGF-C we next asked if this would increase the permeability of the LEC monolayer. Over an 8-hour time course we found an incomplete, but significant, increase in permeability of the LEC monolayer when rVEGF-C was added to the oxLDL treated group (Figure 3B). Similarly, upon plating cells and staining for VE-Cadherin we noticed, as before (Burchill et al., 2021), that VE-Cadherin junctions appear less permeable (continuous/straight line staining of VE-Cadherin between cells) upon oxLDL treatment compared to untreated cells whose junctions appear more permeable (discontinuous/jagged VE-Cadherin staining between cells) (Figure 3C). Indeed, when cells were treated with VEGF-C and oxLDL, as in Figures 3A,B, the junctions appeared more like the vehicle control. The change in VE-Cadherin organization correlated with VE-Cadherin expression as the mean intensity of VE-Cadherin was significantly diminished in the oxLDL group with VEGF-C compared to the oxLDL alone group (Figure 3D). Thus, VEGFR-3 signaling leads to partial rescue of VE-Cadherin junctional proteins on the membrane correlating with a partial rescue of the functional permeability of an LEC monolayer.

### VEGF-D improves oxLDL induced lymphatic dysfunction *in vivo*

**FIGURE 4:**
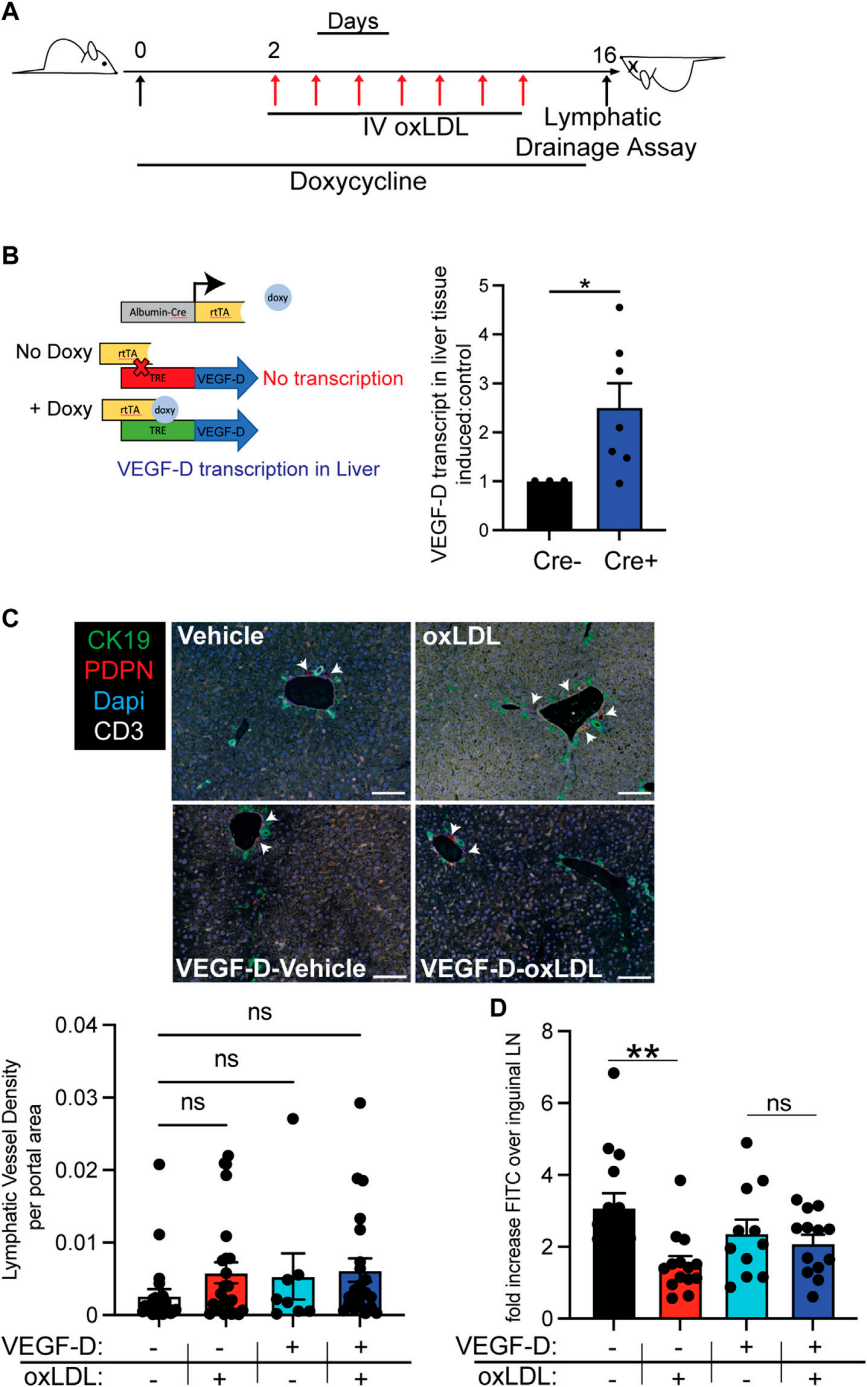
VEGF-D improves oxLDL induced lymphatic dysfunction *in vivo.* **(A)** Experimental design of FITC-dextran assay (as described in Figure 1), VEGF-D expression was induced with doxycycline 48 h and continued during 2-week duration of every other day oxLDL injection with last injection 48 h prior to harvest. **(B)** Genetic model of TRE-VEGF-D x Albumin-Cre promotor rtTA mice developed for hepatocyte-specific activation and expression of excess VEGF-D. Transcript levels of VEGF-D expression in whole liver tissue of control mice (cre-) compared to VEGF-D induced mice (cre+) expressed as fold change from expression level of Cre-mice. Shown is a representative experiment. Experiment was performed 3 times with similar results. **(C)** Lymphatic vessel density as imaged with Perkin Elmer Vectra fluorescent microscope. Bile ducts [CK19 + PDPN+(green)], lymphatics [CK19-PDPN+ (red)] (highlighted with white arrows), T cells [CD3 (white)] and nuclear [Dapi (blue)] were stained and lymphatic vessel density was quantified per portal region from each tissue section in ImageJ [as previously described (Burchill et al., 2021)]. A one-way anova statistical analysis and student’s t-test demonstrated no significant difference among groups. Scale bar is 50 μm. Shown is LVD per portal area from 2-5 mice. Analysis was completed from 2 separate experiments with 2-5 mice per group. **(D)** FITC fluorescence from extracted (liver-draining) portal over (skin-draining) inguinal lymph node 10 min after intrahepatic injection of 500 kDa FITC-dextran. Statistical analysis demonstrated a significant difference between wild-type treated with PBS vs. oxLDL with a *p*-value <0.01 but no significant difference between with VEGF-D inducible mice treated with PBS vs. oxLDL. This experiment was repeated three times with similar results and 3-5 mice per group. Shown is combined data from all 3 experiments. *p < 0.05, **p < 0.01.

As induction of VEGFR-3 signaling in primary human LECs rescued expression of VE-Cadherin and permeability of LECs in response to oxLDL, we next asked if VEGFR-3 signals could restore lymphatic transport from the liver after treatment with oxLDL (Figure 4A). To do this, we utilized a mouse model with hepatocyte-specific inducible overexpression of VEGF-D. As previously published, this mouse permits inducible expression of VEGF-D in a cell-specific manner upon administration of doxycycline (Lammoglia et al., 2016). To induce expression within the liver, these mice (TRE-VEGF-D), were crossed to albumin-Cre x Rosa26STOPfl-rtTA mice (Figure 4B). Following 2 weeks of doxycycline treatment in the water there was an average increase in VEGF-D expression that was 2.5-fold higher in the liver of albumin-Cre x Rosa26STOPfl-rtTA x TRE-VEGFD mice compared to wild-type mice treated with doxycycline (Figure 4B). As VEGF-D induces lymphangiogenesis, we first asked if there was an increase in lymphatic vessel density within the liver following treatment with doxycycline. Likely due to the short duration of VEGF-D in the liver, we did not detect a significant increase in lymphatic vessel density across groups treated with VEGF-D (Figure 4C). Additionally, there was no marked histologic difference of hepatic architecture between groups (Supplementary Figure S4A) nor significant difference in histologic liver injury scoring, with no liver injury induced by oxLDL as compared to models of chronic liver diseases (Supplementary Figure S4B). To determine the effect of VEGF-D overexpression on liver lymphatic function, WT or albumin-Cre x Rosa26STOPfl-rtTA x TRE-VEGF-D inducible mice were given doxycycline in their drinking water for 16 days. Two days following introduction of doxycycline mice were injected (IV) with either oxLDL or PBS over the course of 14 days while doxycycline was continued (Figure 4A). To determine if VEGF-D overexpression in the liver could improve oxLDL-induced liver lymphatic dysfunction we assessed FITC Dextran transport, at 10 min after intrahepatic injection, from the liver parenchyma to the liver draining lymph node as in Figure 1. In these studies, we found that the VEGF-D overexpression during treatment with oxLDL increased the amount of FITC-Dextran reaching the liver draining lymph nodes to a level similar to albumin-Cre x Rosa26STOPfl-rtTA x TRE-VEGFD mice treated only with vehicle (PBS) (Figure 4D). Together these data demonstrate that VEGF-C or VEGF-D administered with oxLDL can increase the amount of FITC Dextran in the liver draining lymph nodes without significant lymphangiogenesis (Figure 4C), consistent with reorganization of lymphatic junctions rather than increased lymphatic vessel density *in vivo*.

### VEGF-D reduces VE-Cadherin expression on liver lymphatics *in vivo*

**FIGURE 5:**
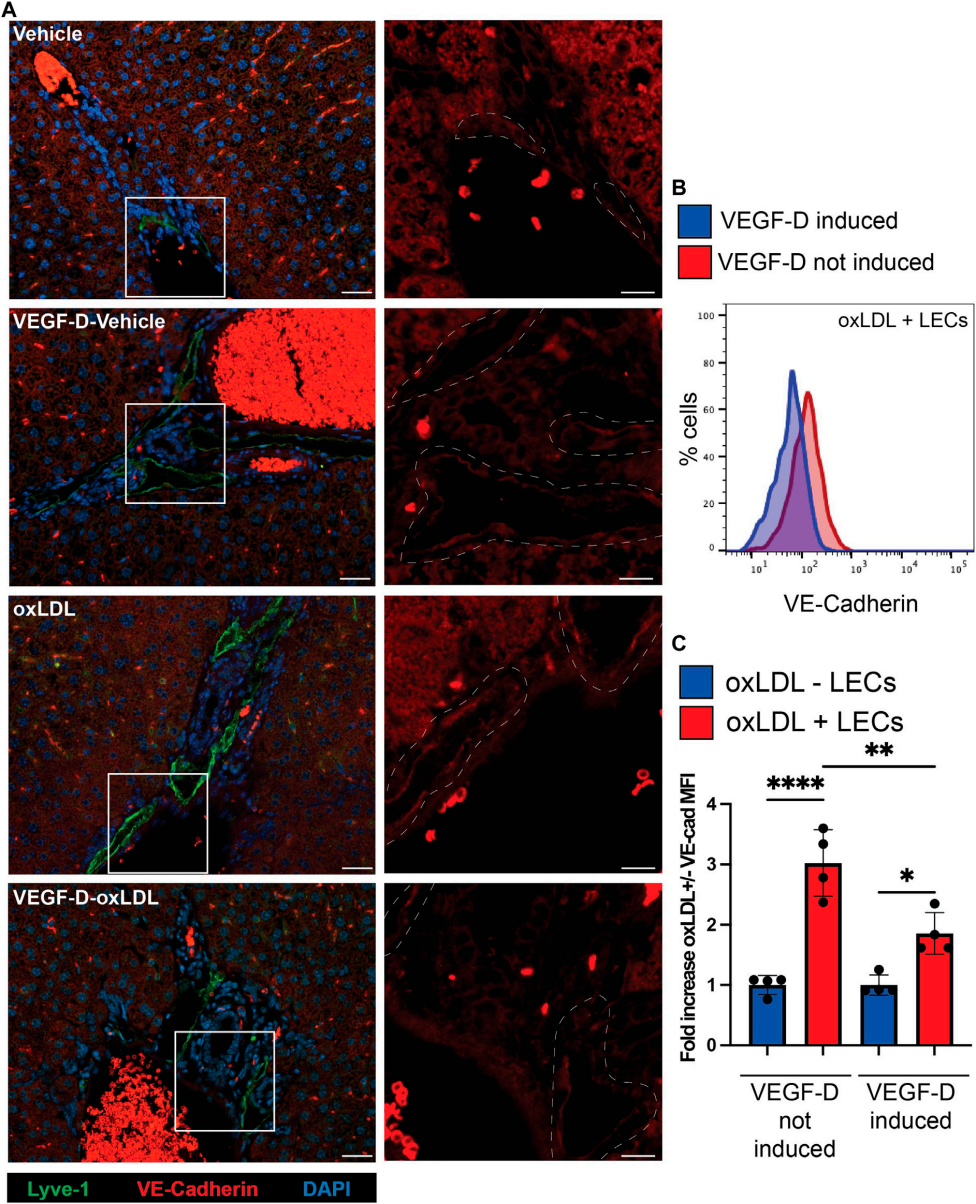
oxLDL increased VE-Cadherin in liver lymphatics is decreased by VEGF-D induction *in vivo*. **(A)** Mice were treated exactly as in Figure 4A. Non-induced mice were wild-type mice treated with doxycycline, VEGF-D mice were TRE-VEGF-D mice treated with doxycycline. Mice were injected with either PBS or oxLDL as described in methods. Liver tissue sections were stained with VE-Cadherin (red), Lyve-1 (green) and Dapi (blue). Scale bars on zoomed out images are 20 μm and scale bars on zoomed in VE-Cadherin only images are 10 μm. Dashed white lines indicate areas of lyve-1/lymphatic staining. 3-5 liver sections from 2 experiments were stained and imaged. **(B)** TRE-VEGF-D mice treated with doxycycline water for 2 days prior to oxLDL-DIL injection. 4–6 days later livers from euthanized mice were digested and made into a single cell suspension prior to staining for lymphatic endothelial cells. Shown is CD45^−^CD31^+^CD146-PDPN^hi^oxLDL + cells VE-Cadherin expression between VEGF-D induced (blue) and VEGF-D not induced (red). **(C)** Quantification of oxLDL+ and oxLDL- LEC expression of VE-Cadherin. VE-Cadherin expression in oxLDL-cells was normalized per group and fold increase over each control was quantified. Experiment was repeated twice with 3-4 mice per group with similar results. *p < 0.05, **p < 0.01, ****p < 0.0001.

We next asked if lymphatics in the liver also upregulated VE-Cadherin in response to oxLDL. To do this we evaluated liver tissue sections of mice given oxLDL or PBS in the albumin-Cre x Rosa26STOPfl-rtTA x TRE-VEGF-D from Figure 4. Sections were stained with Lyve-1 (lymphatics) and VE-Cadherin. Portal areas from each group were imaged (Figure 5A). As expected, the oxLDL treated mice from the non-induced control group appeared to have increased staining of VE-Cadherin (red) in the lymphatics identified by Lyve-1 (green) (Figure 5A). This increased intensity of VE-Cadherin on liver lymphatics only appeared in the oxLDL injected non-induced control mice while all other groups appeared relatively similar. To quantitatively measure the differences in VE-Cadherin expression, we next evaluated VE-Cadherin levels on LECs using flow cytometry. Mice were injected with fluorescently labeled oxLDL (DIL) 4–6 days prior to euthanasia. We identified LECs from the liver that had acquired the oxLDL using the fluorescent label (Supplementary Figure S5) as previously described (Tamburini et al., 2019). Upon evaluation of LECs that were positive for oxLDL we found that VE-Cadherin expression was increased in non-induced control mice compared to mice in which VEGF-D was induced (Figure 5B). When we quantified the increase in VE-Cadherin expression, we found that in both cases oxLDL + LECs had increased expression of VE-Cadherin when compared to oxLDL- LECs. However, we found significantly lower VE-Cadherin expression by oxLDL + LECs from the livers of VEGF-D induced (Figure 5C). Together, these findings provide evidence that levels of VE-Cadherin increase, not only in *in vitro* treated human lymphatic endothelial cells, but also in the liver lymphatics following exposure to oxLDL which can be mitigated by increasing VEGF-D levels.

## Discussion

Our study demonstrates that lymphatic drainage is impaired across multiple modalities of liver disease (Burchill et al., 2021). While we have previously shown that lymphatic vessel density in the liver is increased across human liver diseases and murine models of NAFLD, ALD and cholestasis, the findings reported here challenge the idea that increased lymphatic vessel density in disease leads to increased transport of protein to the lymph node. Increased lymphatic vessels in disease may attempt to accommodate for excess fluid and protein transport, but our findings demonstrate that a lymphatic adherence junction protein, VE-Cadherin, is increased which prevents high molecular weight dextrans from entering the draining lymph node after exposure to inflammatory oxLDL. We believe that this defect in lymphatic drainage and mis-regulation of VE-Cadherin could be due to the sprouting of new lymphatic capillaries in a setting of inflammation. Newly sprouted lymphatic capillaries have tight “zipper-like” junctions rather than “button-like” junctions (Baluk et al., 2007), therefore it is possible that the new lymphatic capillaries maintain zipper-like junctions in the presence of oxLDL. Our data would suggest that VEGF-C or D can counteract this maintenance of zipper like junctions. While we do show a direct effect of oxLDL on the lymphatic endothelial cells both *in vitro* and *in vivo* in the upregulation of VE-Cadherin, it is also possible that other cells contribute to the defect in lymphatic drainage. As an example, we and others have demonstrated increased numbers and activation status of macrophages in the livers of *mdr2−/−*, Lieber-Decarli, and high fat high cholesterol fed mice (Wang et al., 2014; Guicciardi et al., 2018; McGettigan et al., 2019; Ceci et al., 2022). Reduced protein transport to the portal lymph node during chronic disease appears to be a common feature among chronic liver diseases associated with high oxLDL, at least in mice. However, our murine data, combined with the similarities in architectural changes in human liver disease and decreased protein content in lymph during cirrhosis (Dumont and Mulholland, 1960; Dumont and Mulholland, 1962), strongly supports the conclusion that lymphatic transport of protein is impeded in people with chronic liver disease. With the important role of lymphatics in fluid and protein homeostasis as well as immune surveillance it seems likely that this lymphatic disruption is contributing to the pathogenesis of these liver diseases.

While changes in blood vascular endothelial, specifically sinusoidal defenestration, in the liver has previously been described and studied in chronic liver diseases (Zhang et al., 2014), the lymphatics remain understudied. Here we highlight that the lymphatic vasculature is uniquely altered in response to oxLDL. Specifically, we found that *in vitro* permeability was decreased only in LECs as compared to LSECs. We found intracellular signaling in response to oxLDL to differ between LECs and LSECs with increases in phosphorylation of SRC family kinases and p38, a MAPK suggested to be downstream of SRC family kinases (Maneechotesuwan et al., 2007; Maneechotesuwan et al., 2009). Phosphorylated P38 and SRC have also been associated with CD36 and VEGFR-2 signaling. As these signaling pathways are active after oxLDL treatment of LECs, we speculate that oxLDL scavenger receptor, CD36, signaling may regulate VE-Cadherin and/or VEGF receptor levels. Furthermore, as lymphatic paracellular permeability but not liver sinusoidal endothelial permeability is regulated by VE-Cadherin *in vitro*, we conclude that reduced FITC Dextran transport from the liver to the liver draining lymph node during disease or oxLDL treatment is a result of lymphatic, rather than sinusoidal, endothelial changes. These findings are consistent with permeability of LSECs *in vivo* being regulated by fenestrae rather than cell-cell junctions (Szafranska et al., 2021), where *in vivo* VE-Cadherin, N-Cadherin, E Cadherin and Claudin 5 expression by LSECs maintains tight cell-cell junctions, similar to other blood vasculature endothelial cells (Zhang et al., 2018). This is particularly important as we dissect the contribution of LSEC versus LEC in response to oxLDL in the liver and demonstrate consistent changes in lymph transport across disease etiologies where liver concentrations of oxLDL are high.

In our previous study the ratio of VEGFR-2/VEGFR-3 was increased both in LECs in the livers of diseased mice and in LECs treated with oxLDL *in vitro* (Burchill et al., 2021). This suggested a similar mechanism as described in another study where increased VEGFR-2 signaling led to decreased permeability and rearrangement of the VE-Cadherin junctions (Zhang et al., 2020). In this study we demonstrated that we could normalize the transcript ratio of VEGFR-2/VEGFR-3 with rVEGF-D. This treatment with VEGF-D also resulted in a reduction in VE-Cadherin gene and protein expression following oxLDL treatment, suggesting that VEGFR-2/3 levels may significantly influence VE-Cadherin levels. VE-Cadherin surface expression was also decreased by the addition of rVEGF-C (cys156ser) in LECs in response to oxLDL. This regulation of VE-Cadherin expression by VEGF-C correlated with increased permeability of LECs in the presence of oxLDL. However, as we only detected a partial decrease of VE-Cadherin and a partial increase in permeability there may be additional factors that contribute to VE-Cadherin expression and the resulting differences in permeability in response to oxLDL.

Here we show that differences in lymphatic permeability and function *in vitro* can be modeled experimentally in mice. As such, excess intrahepatic VEGF-D also prevented oxLDL induced loss of lymphatic transport of FITC Dextran. These new findings are in line with our previous studies demonstrating that lymphatic transport of FITC Dextran in a murine model of NASH can be rescued with exogenous administration of VEGF-C after steatohepatitis has already been established (Burchill et al., 2021). Our findings are consistent with the ability of excess VEGF-C to ameliorate defects in lymphatic drainage, excess portal pressure and ascites in experimental portal hypertension (Kaur et al., 2021). The importance of these studies in modeling human disease is emphasized by the finding that genetic defects in VEGF-C correlates with increased risk of variceal bleed as a result of portal hypertension in HCV patients with end stage liver disease (Aboismail et al., 2022).

In conclusion, oxLDL is a common mediator of chronic liver diseases that disrupts LEC cell-cell junctions by affecting LEC signaling, an effect that can be at least partially rescued by excess lymphatic-specific VEGF-C/D. These studies are important because lymphatic function in liver diseases has been understudied and we have previously demonstrated that restoration of lymphatic drainage can reduce inflammation in a murine model of NASH (Burchill et al., 2021). Despite the lack of information about lymphatics in the liver, there are several anecdotal pieces of evidence that suggest improving lymphatic function in the liver may be a viable target to slowing and/or reversing the progression of liver disease. There are repeated indications that lymphatic dysfunction and loss of lymphatic VEGF-C signaling results in exacerbation of clinical findings of liver disease including portal hypertension and ascites (Kaur et al., 2021; Aboismail et al., 2022). Furthermore, VEGF-C treatment in atherosclerosis, another disease where oxLDL is thought to contribute, improves lymphatic function and decreases plaque formation (Silvestre-Roig et al., 2021). Finally, as our present study has clearly demonstrated a link between oxLDL, VE-Cadherin, VEGFR signaling and impairment in LEC permeability, our future studies aim to elucidate which oxLDL receptors are important for LECs, how oxLDL disrupts VEGFR and VE-Cadherin signaling, and whether there are more specific targets that can be harnessed for potential therapeutic options.

## Materials and methods

### Animal studies and feeding regimens

Experiments utilized 6- to 8-week-old male or female C57BL/6 mice purchased from Charles River Laboratories (Wilmington, MA) or Jackson Laboratories (Bar Harbor Maine). For diet studies, male mice were randomly allocated to either a chow control or HFHC diet (#D17010101-03) formulated by Research Diets Inc. (New Brunswick, NJ) as previously described (McGettigan et al., 2019). Mice were provided diet *ad libitum* for a period of 20–24 weeks. For Mdr2 knockout mice, mice were bred on a C57BL/6J background and male or female mice aged to 10–14 weeks prior to our studies. The mice were provided by the laboratory of Dr. Sean Colgan. For chronic ethanol fed mice, female animals were randomized into ethanol-fed and pair-fed groups, adapted to control liquid diet for 2 days and fed increasing concentrations of ethanol up to 6% v/v (32% kcal) for 25 days as previously described (Cohen et al., 2010). For treatment studies, male or female of 6–10 weeks of age mice were injected (intravenously) with 150 μg of highly oxidized LDL (Kalen Biochemicals, Germantown, MD) or phosphate-buffered saline (PBS) as a vehicle control in a total volume of 200 μL. Tetracycline-response element (TRE) promoter VEGF-D mice (TRE–VEGF-D) were generated as described (Lammoglia et al., 2016). The TRE-VEGF-D mice were then crossed mice with Rosa26STOPfl-reverse (tetracycline–dependent trans activator (rtTA)) mice. These TRE-VEGF-D x Rosa26STOPfl-rtTA (JAX:005572) were then crossed with albumin-Cre mice (JAX:003574) to produce albumin-Cre x Rosa26STOPfl-rtTA x TRE-VEGF-D mice for hepatocyte specific activation of VEGF-D. Albumin-cre+ or − x Rosa26STOPfl-rtTA x TRE-VEGF-D or WT mice were provided water with doxycycline (200 mg/L) daily for 2 days prior and continued through course of oxLDL injections. Injections of oxLDL or vehicle occurred twice weekly for a period of 2 weeks with last injection 48 h prior to mouse harvest. For *in vivo* analysis of VE-Cadherin expression by flow cytometry, 125 ug of DIL-labeled oxLDL (Kalen Biochemicals) was I.V. injected into albumin-cre+ x Rosa26STOPfl-rtTA x TRE-VEGF-D mice with or without VEGFD induction as described above, and livers were harvested for flow cytometry 4–6 days post injection. All animal studies were approved by the University of Colorado Anschutz Medical Campus Institutional Animal Care and Use Committee.

### Liver lymphatic drainage assay

Mice were anesthetized with a solution of ketamine (30–60 mg/kg) and xylazine (3–6 mg/kg) and placed on a heating pad. An incision was made into the peritoneum to expose the liver and 5–10 μL of a solution of 12.5 mg/ml of FITC-labeled dextran (500 kDa) in PBS was injected into left, median, and right lobes of the liver using a 28-gauge needle (BD Biosciences, San Jose, CA). Ten minutes after FITC-dextran administration, the liver-draining lymph node (portal) were excised and placed into separate wells containing 400-μL cold PBS. As a control for vascular drainage of the dextran, the inguinal (skin draining) lymph node was also excised and placed in a separate well with PBS. Lymph Nodes were minced with 22-gauge needles, and 200 μL of the minced LN in PBS was placed in a 96-well Costar Assay Plate (Corning, Corning, NY), and FITC was read using a Synergy H1 microplate fluorescence plate reader (BioTek, Winooski, VT). Data were normalized to the fluorescence of the inguinal lymph node from the same mouse taken immediately after the portal LN.

### Semi-quantitative PCR

Briefly, snap-frozen liver tissue or cultured cells were homogenized in Buffer RLT or RLT + BME and total RNA was isolated from cell lysate using the RNeasy Mini Kit (Qiagen, Hilden, Germany), and complementary DNA was synthesized using the QuantiTect RT Kit (Qiagen) following standard protocols. PCR amplification was performed using either the QuantiTect Sybr green (Qiagen) or TaqMan Fast Advanced Master Mix (Applied Biosystems, Foster City, CA) PCR kits. Quantitative PCR was performed on a QuantStudio 3 Real-time PCR machine (Applied Biosystems, Waltham, MA) and fold changes in messenger RNA levels were calculated. For each gene, all samples were normalized to the average fold change of the control treatment group (chow, WT, pair-fed, or PBS). The following Qiagen QuantiTect primer assays were used: 18S ribosomal RNA (Rn18s; QT02448075), CD36 (QT01058253); VEGF-D/FIGF (QT QT00164024) for mouse.

### *In Vitro* permeability assay

10,000 human lymphatic endothelial cells (hLECs) (PromoCell, Heidelberg, Germany) or human liver sinusoidal endothelial cells (hLSECs) (Sciencell, Carlsbad, CA) were plated on a 0.2% gelatin–coated 24-well Costar Transwell permeable support (6.55-mm insert) with a 0.4 mm Polyester Membrane (Corning) and grown for 3 days. In experiments with supplemental VEGF treatment, cells were treated in serum-free media a total of 60 h after plating cells and treated with PBS or recombinant human VEGF-C (cys156ser, 0.1ug/mL) (R&D systems, Minneapolis, MN) for 12 h before the addition of oxLDL (Kalen, 100ug/mL) for 4 h for a total of 16 h of treatment with rVEGF-C prior to the addition of FITC-dextran. For cells treated with oxLDL (Kalen, 10 or 100ug/mL) or Anti-CD144 Mouse Monoclonal Antibody (VE-Cadherin clone BV9, Biolegend, San Diego, CA) cells were treated in serum-free media for a total of 4 h prior to the addition of FITC-dextran. Following incubation, 500-kDa FITC Dextran (1.25 mg/ml) in Hanks’ balanced salt solution was added to the top of the well. Media were removed from the bottom of the transwell and replaced with HBSS. Plates were incubated at 37°C with gentle rocking and migration of the FITC-labeled dextrans was measured using a Synergy H1 microplate reader by removing 60 μL from the bottom of the transwell at indicated time points at an excitation of 485 nm and emission of 528 nm. Fluorescence readings were normalized to the average of the well with PBS treatment alone at the 1-hour timepoint within the experiment.

### Western blots

hLECs or hLSECs were grown to >80% confluency in T25 flasks then treated with oxLDL at 100 μg/ml for either 0, 0.5, 4 or 24 h. Treated as well as untreated cells were washed with cold PBS and cells were coated with 500 μL of lysis buffer (from RayBiotech kit AAH-MAPK-1-2 prepared with supplied protease and phosphatase inhibitors to manufacturer instructions). Lysate was collected and clarified by spinning at 12,000 rpm for 20 min at 4°C. Supernatant was collected and quantified using BCA kit (Thermo Fisher Scientific, Waltham, MA). Gel samples were made by combining equal amounts of total protein from each sample diluted to equal volume with lysis buffer. 4x Laemmli buffer with 10% 2-mercaptoethanol was added for a final concentration of 1x, then samples were heated on a heat block of 90°C for 10 min. Samples were run on 10% acrylamide gels and transferred to 0.45-μm polyvinylidene difluoride membrane. Membranes were blocked with 5% BSA/Tris-buffered saline with 0.1% Tween 20 detergent [TBST] for 30–60 min at room temperature while rocking. Then membranes were incubated in anti-VE-cadherin (AB33168 Abcam), anti-SRC (S1208S Cell Signaling), anti phosphor Src Family (Tyr416, 6943S Cell Signaling), anti P38 MAPK (8690S Cell Signaling), anti-Phospho-p38 MAPK (4511S Cell Signaling) or anti-GAPDH-HRP (MA5-15738-HRP; Invitrogen, Carlsbad, CA) diluted 1:1000 or 1 μg/ml in 5% BSA/TBST or anti-Tubulin (AB6160 Abcam) diluted 1:10,000 or 1ug/mL in 5% BSA/TBST overnight at 4°C while rocking. Membranes were then washed with TBST 3 × 5 min then for VE-Cadherin incubated with anti-Rabbit-HRP (AB7090 Abcam 1:10,000 in 5% BSA/TBST) or anti-Rat-HRP for the Tubulin (ab97057 Abcam 1:10,000 in 5% BSA/TBST) at room temperature for 1 h while rocking. Membranes were then washed in TBST and imaged using Pierce ECL western blotting substrate and the ChemiDoc MP imaging system (Bio-Rad, Hercules, CA). Quantification of Western blot was performed using ImageLab (Bio-Rad) analysis software. Pixel intensity was quantified for each band and then normalized to the loading control for each individual sample before dividing the values by the time zero values in order to obtain the fold increase over vehicle (t = 0).

### Flow cytometry

Flow cytometric analysis of cultured cells: hdLECs (Promocell) and hLSECs (Sciencell) were cultured in 6-well tissue-coated plates (Corning). When cells reached 70–80% confluency, cells were treated with rVEGF-C (cys156ser, R&D Biosystems) for 12 h and then the addition of highly oxidized low-density lipoprotein (Kalen) at concentration of 100 μg/ml for 4 h for a total of 16 h of treatment in serum-free media. Cells treated with oxLDL alone were treated at concentrations of 10 or 100 μg/ml oxLDL (Kalen) for 4 h total in serum-free media. Cells were then detached from plate with Promocell detach kit (Promocell) and stained with anti-CD144 Mouse Monoclonal Antibody in PE (BioLegend, San Diego, CA) Human Fc Block (Fisher Scientific, Hampton, NH) for 30 min at 4°C. Following staining, cells were washed and then run on the flow cytometer. All samples were collected on a BD LSR canto II flow cytometer using DIVA software (BD Biosciences) and analyzed with FlowJo software (Treestar, Ashland, OR).

Flow cytometric analysis of liver endothelial cells: Non-parenchymal cells were isolated from livers of treated mice as previously described (Burchill et al., 2021). Briefly, livers were extracted and digested with collagenase type IV and NPCs were isolated with a 26% Optiprep (Sigma) density gradient. Enriched NPCs were stained with the following antibodies CD31 (390), CD45 (30-F11), CD144 (BV13), and PDPN (8.1.1) from Biolegend and CD146 (ME-gF1) from BD Biosciences. Samples were collected on a flow cytometer as above.

### Immunofluorescence, microscopy, and liver histology

Liver tissue was explanted from the mouse and liver sections fixed in 10% formalin. Sections were cut in 5-μm-thick sections and adhered to a glass slide. Slides were dewaxed with xylene, heat-treated in either pH 6 or pH 9 antigen retrieval buffer for 15 min in a pressure cooker, and blocked in antibody diluent (PerkinElmer, Waltham, MA). Sections were then sequentially stained for CD3 (ab699), PDPN (clone 8.1.1, BioLegend 127407), and CK19 (ab52625 primary antibodies followed by HRP-conjugated secondary polymer (anti-rabbit [PerkinElmer], anti-goat ab97110, or anti-hamster ab6892) and HRP-reactive OPAL fluorescent reagents (PerkinElmer). To image nuclei, slides were stained with spectral DAPI and coverslips were applied with Prolong Diamond mounting media (Thermo Fisher Scientific). Whole slide scans were collected using the ×10 objective and 5–10 regions were selected for multispectral imaging with the ×20 objective. The multispectral images were analyzed with inForm software (Perkin Elmer) to unmix adjacent fluorochromes, subtract autofluorescence.

For VE-Cadherin staining of liver sections: liver sections from TRE-VEGFD or WT mice, that were treated with doxycycline to induce VEGFD expression from hepatocytes were stained as follows. The slides were baked at 60°C for 2 h and deparaffinized in xylene. The slides were hydrated and antigen retrieval was performed at pH9 (Akoya Biosciences AR9) was done in a pressure cooker on high. Slides were washed (2.5% FBS in PBS for wash buffer, kept on ice) and blocked with 5% goat serum and 5% donkey serum in PBS for 1 h. Slides were then stained with anti Lyve-1 antibody (abcam, ab14917) 1:100, anti CD144 (VE-Cadherin) antibody (biolegend, clone 11D4.1) 1:40 overnight at 4°C. Slides were washed then placed in secondary antibody (PE Goat anti Rat IgG (biolegend, Poly4054) and Alexa Fluor 647 donkey anti-rabbit IgG (H + L), both 1:400). Slides were washed again before being sealed with Vecta shield (Vecta Vibrance, hardset with Dapi) and imaged on a Nikon immunofluorescence microscope in NIS Elements.

For VE-Cadherin staining of cells: VE-Cadherin staining was performed on cells plated on a chamber slide (Thermo Fisher Scientific) and grown to confluence, then treated for 24 h with 100 μg/ml oxLDL (Kalen) and/or rVEGF-D (0.1ug/mL) (R&D systems), then fixed with 2%PFA for 15 min at room temperature. Cells were then rinsed and blocked with 10% donkey serum, 2% BSA, for 1 h at room temperature. Staining was performed with rabbit anti-VE-cadherin 1:100 (Abcam) and DAPI 1:1000 (BioLegend). The filter was removed from the transwell insert and mounted on a microscope slide and read on a Nikon microscope using NIS-Elements.

### Quantification of LVD

LVD is defined as the area of the lymphatics divided by the defined tissue area. For lymphatic density in murine livers, regions portal tracts were selected and images were exported in tiff format after unmixing in Inform software (Akoya Biosciences, Marlborough, MA. Lymphatics were colored in using the color tool and the whole 1800 × 1500 area was opened with ImageJ version 1.52T (National Institutes of Health, Bethesda, MD). The image was converted into an 8-bit image and the threshold was adjusted so only the drawn lymphatic area was seen. The analyze particles function was used to determine the area of the lymphatics. Area of lymphatics in each portal triad was then divided by the area of the portal triad (∼0.7 mm^2^) to calculate lymphatic vessel density per portal area. Approximately 5 portal areas were calculated per liver tissue section (∼70 mm^2^) in 2-5 mice per group from 2 different experiments.

### Statistical analysis

All statistical analysis was performed using a Student’s *t* test or 1-way analysis of variance with multiple comparisons to obtain a *p* value. To assess differences between lines from time-courses of FITC drainage, linear regression analysis was performed to assess significant differences in the slope of the lines. *p* values are designated by asterisks in which ∗*p <* 0.05, ∗∗*p <* 0.01, ∗∗∗*p <* 0.001, ∗∗∗∗*p <* 0.0001. The number of animals used per experiment was determined based on a power calculation. Statistically significant differences between control and experimental groups (*p* ≤ 0.05 with determined number of animals per group) were obtained in at least 2 experiments (1 + 1 repeat) and in most cases 3 experiments. Based on our Institutional Animal Care and Use Committee (IACUC) protocol, unnecessary animal experiments were not performed. For *in vitro* experiments, all experiments were performed a minimum of 3 independent times with 3–5 replicates per group. All authors had access to the study data and had reviewed and approved the final manuscript.

## Data Availability

The raw data supporting the conclusions of this article will be made available by the authors, without undue reservation.
