# Supplementary material for: Oxidized low density lipoprotein in the liver causes decreased permeability of liver lymphatic- but not liver sinusoidal-endothelial cells via VEGFR-3 regulation of VE-Cadherin
Source: Front Physiol. 2022 Oct 19;13:1021038. doi: 10.3389/fphys.2022.1021038 (PMC9626955; doi:10.3389/fphys.2022.1021038)
Supplement: Supplementary file 1 [file DataSheet1.docx]

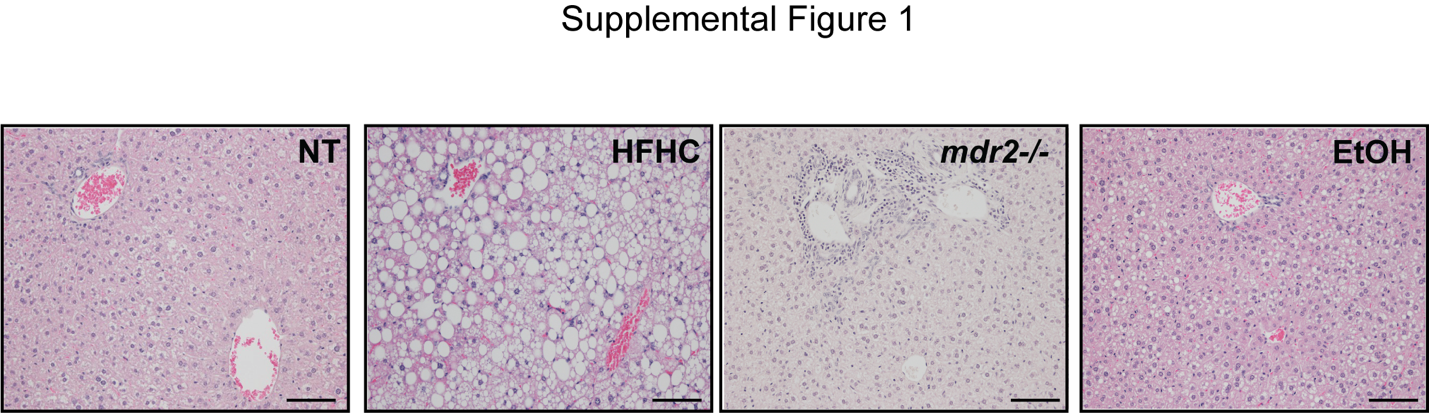


Supplemental Figure 1. Representative images of hematoxylin and eosin-stained hepatic tissue from control, 24-week HFHC diet mice, *Mdr2 -/-* mice and Lieber de Carli mice. Scale Bar is 100μm.


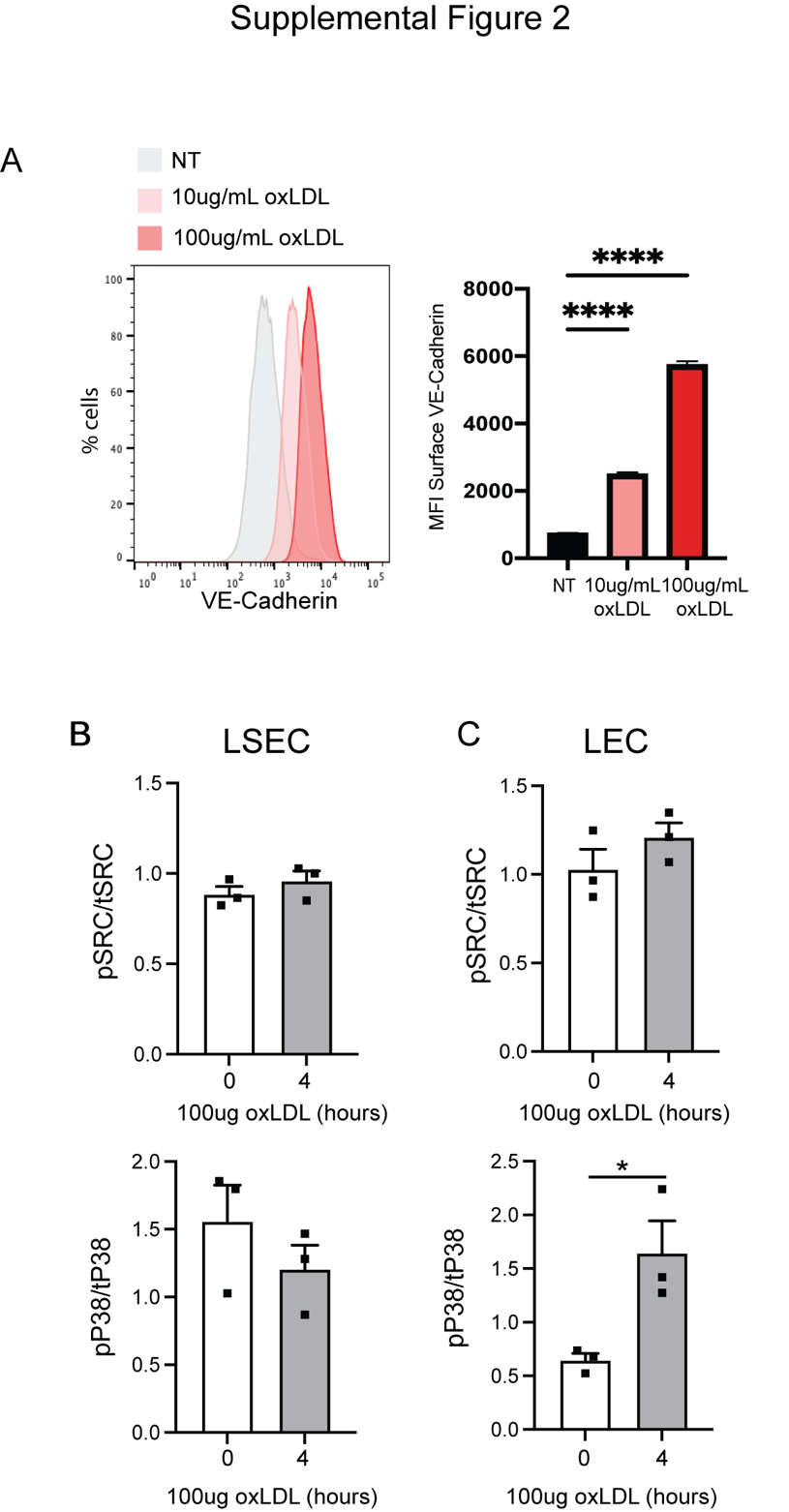


Supplemental Figure 2. VE-Cadherin flow cytometry

A. Histogram of VE-Cadherin surface expression on LECs treated with 0, 10μg/mL or 100μg /mL oxLDL for 4 hours before harvest and staining with BV9 clone of VE-Cadherin directly conjugated to pacific blue. Bar graph representative of gMFI of surface VE-Cadherin in LECs with above treatments and ANOVA analysis demonstrated in a significant difference between NT and both low and high dose oxLDL. B. Quantification of LSEC Western blots from figure 2G normalized to non-phosphorylated proteins. C. As in B, but with LECs. This experiment was repeated three times with similar results. *p<0.05, ****p<0.0001.


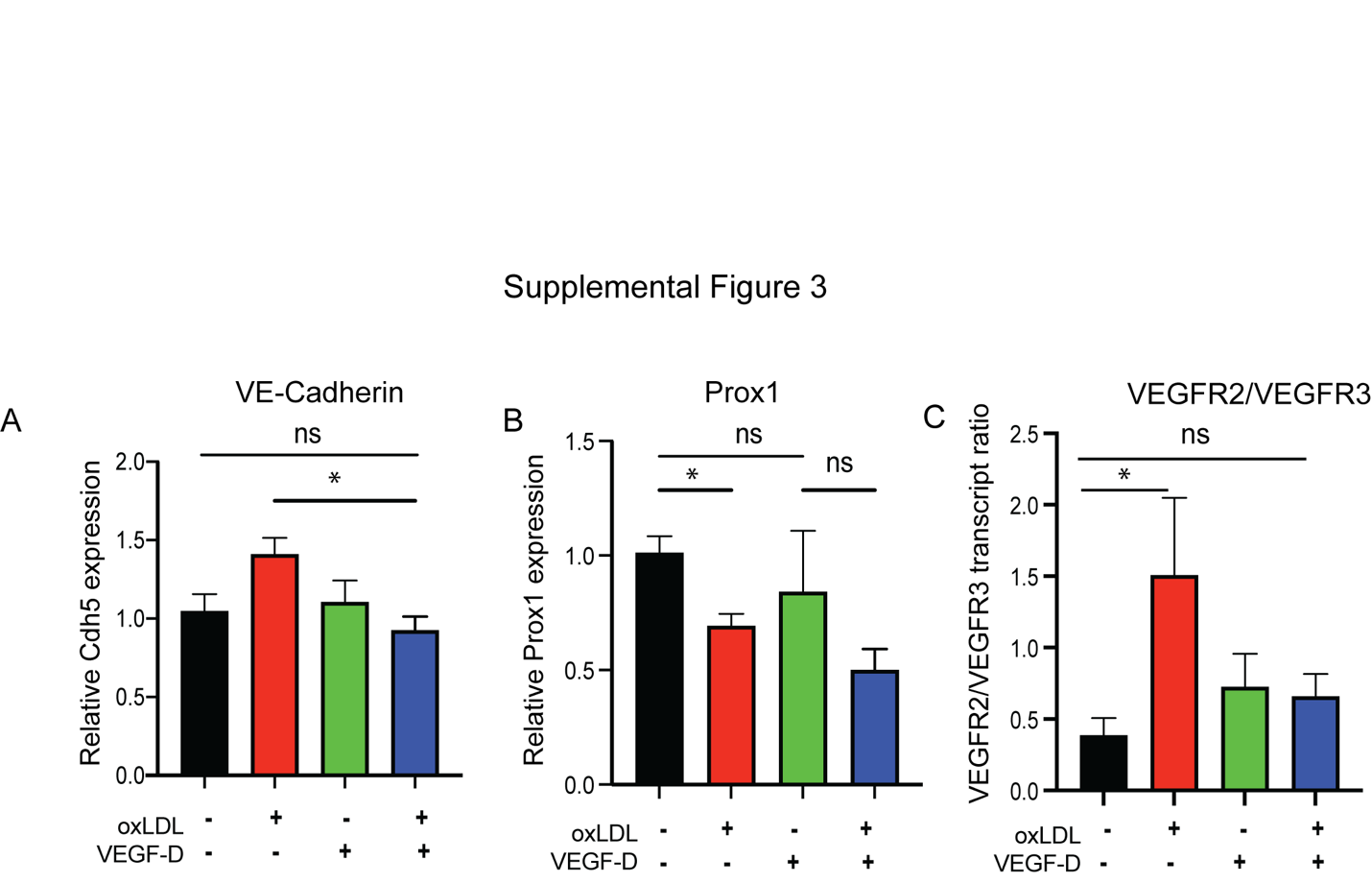


Supplemental Figure 3. Transcriptional profiles of LECs after treatment with **A.** hLECs allowed to grow to 50% confluency then treated with 100ug/mL oxLDL and/or 500ng/mL rVEGFD or PBS in serum free media for 24 hours after which they were harvested and RNA extracted. RT-qPCR transcript for VE-Cadherin as normalized to GAPDH transcript and represented as fold change from PBS treated cells. P-value between oxLDL and oxLDL+VEGFD is .05 as represented by one star with no significant difference between NT and oxLDL+VEGFD groups. Experiment was repeated twice with similar results **B.** Prox1 transcript levels as above. **C.** VEGFR2/3 transcript ratio in hLECs treated with PBS, VEGFD, oxLDL or oxLDL with VEGFD for 24 hours. VEGFR2 and VEGFR3 normalized expression compared to no treatment in LECs as described in A. Experiment repeated twice with similar results.


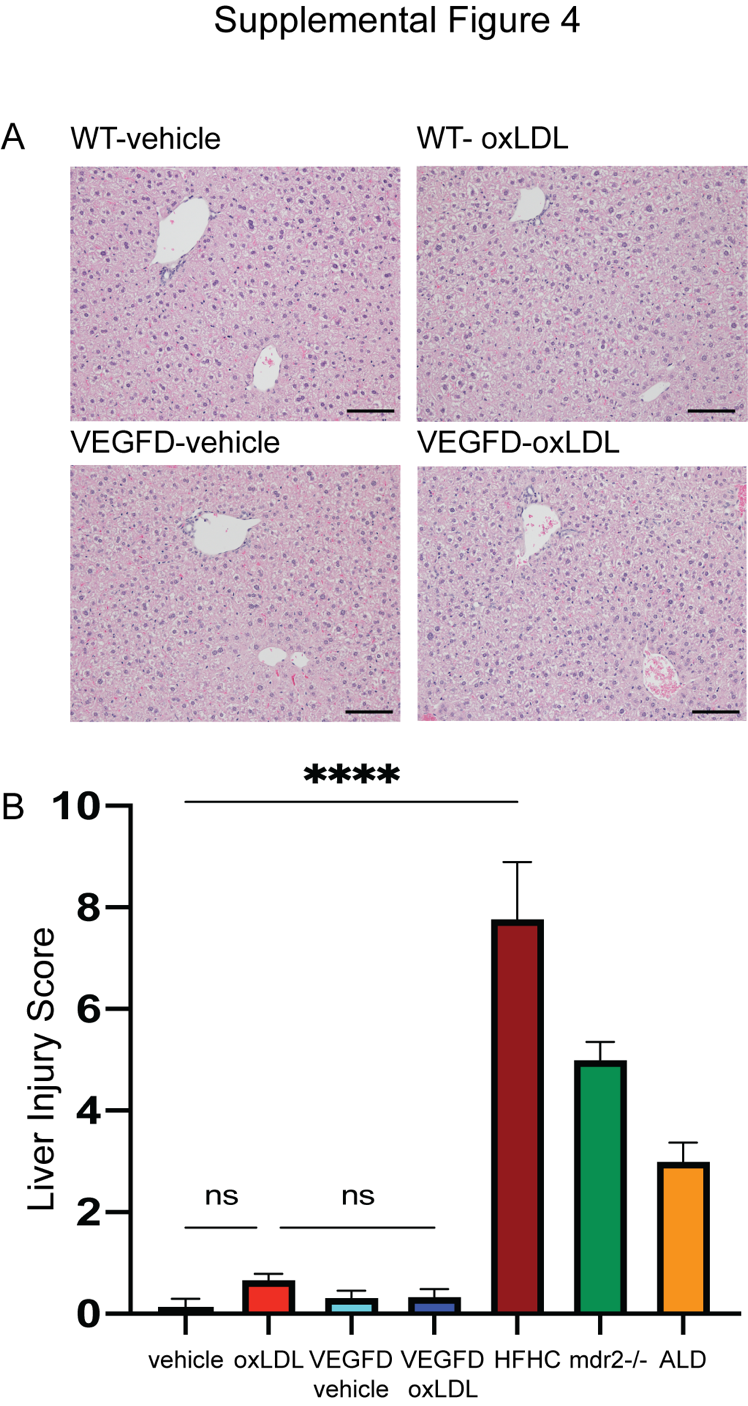


Supplemental Figure 4. Liver Pathology of TRE-VEGFD mice.

**A**. Sample images of liver tissue from livers harvested from experiment described in Figure 4 with Cre+ mice demonstrate no significant architectural changes between treatment groups. Scale bars 100 μm. **B**. Liver histology scoring accounting for cell injury, steatosis, reactive changes and inflammatory infiltrate on histology as compared between experimental groups demonstrating no significant injury in TRE-VEGFD nor WT mice treated with oxLDL. Significant liver injury in all three models of chronic liver disease described above.


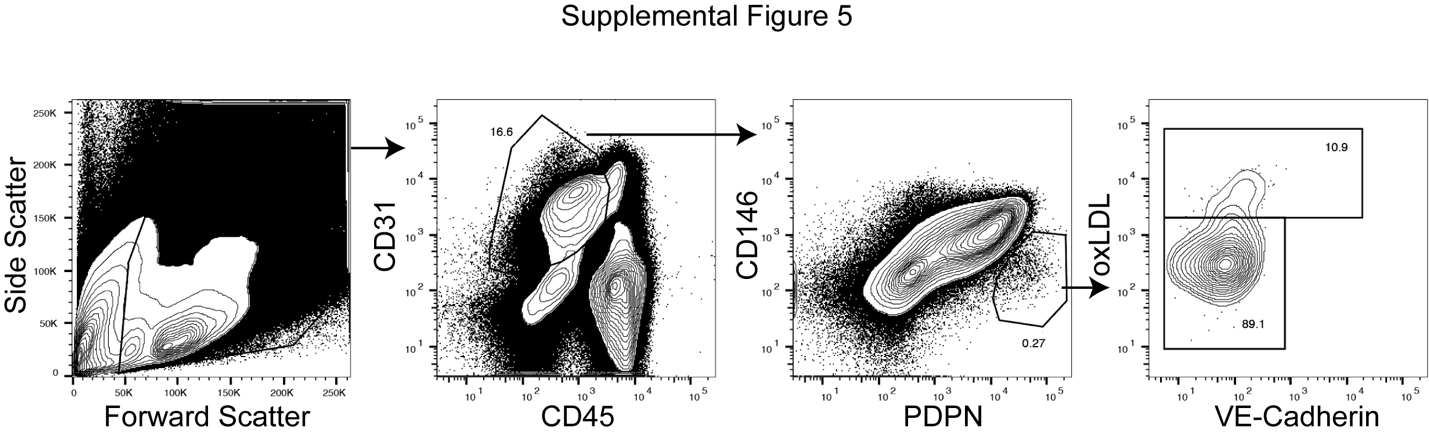


Supplemental Figure 5. Gating strategy for LECs using flow cytometery.

**Supplemental Materials and Methods (not addressed in main body)**

Quantification of Liver Pathology

For quantification of liver histology, liver sections were stained with hematoxylin and eosin and scored semi-quantitatively for steatosis and inflammation as previously described. [1]Briefly, liver sections were given a numeric score for each feature within a given pathology category. Within each category individual pathological features were given a numerical score (0–4) based on how prevalent it was. Thus, the total scores for each pathology category (steatosis (max score 5) and injury (max score 14 cumulative from cell injury, inflammation, reactive changes) were simply a sum of the individual scores for each feature. Histology scoring of blinded liver sections was performed by D.J.O., who was blinded to the identity of the liver section being scored.

Semi-quantitative PCR

The following Qiagen QuantiTect primer assays were used for human cells: VE-Cad (CDH5): QT000013244, Prox1 (QT01006670), VEGFR2(KDR) (QT00069818), VEGFR3(FLT4) (QT000063637) GusB (housekeeping gene, QT000046046).
